# Transmission of methicillin-resistant *staphylococcus aureus* in the long term care facilities in Hong Kong

**DOI:** 10.1186/1471-2334-13-205

**Published:** 2013-05-06

**Authors:** Vincent CC Cheng, Josepha WM Tai, Zoie SY Wong, Jonathan HK Chen, Kris BQ Pan, Yizchen Hai, Wing-Chun Ng, Denise MK Chow, Miranda CY Yau, Jasper FW Chan, Sally CY Wong, Herman Tse, Sophia SC Chan, Kwok-Leung Tsui, Felix HW Chan, Pak-Leung Ho, Kwok-Yung Yuen

**Affiliations:** 1 Department of Microbiology, Queen Mary Hospital, Hong Kong Special Administrative Region, China; 2 Infection Control Team, Queen Mary Hospital, Hong Kong Special Administrative Region, China; 3 Department of Systems Engineering and Engineering Management, City University of Hong Kong, Hong Kong Special Administrative Region, China; 4 Community Geriatric Assessment Team, Fung Yiu King Hospital, Hong Kong Special Administrative Region, China; 5 School of Nursing, The University of Hong Kong, Hong Kong Special Administrative Region, China; 6 Carol Yu Centre for Infection, The University of Hong Kong, Hong Kong Special Administrative Region, China

## Abstract

**Background:**

The relative contribution of long term care facilities (LTCFs) and hospitals in the transmission of methicillin-resistant *Staphylococcus aureus* (MRSA) is unknown.

**Methods:**

Concurrent MRSA screening and *spa* type analysis was performed in LTCFs and their network hospitals to estimate the rate of MRSA acquisition among residents during their stay in LTCFs and hospitals, by colonization pressure and MRSA transmission calculations.

**Results:**

In 40 LTCFs, 436 (21.6%) of 2020 residents were identified as ‘MRSA-positive’. The incidence of MRSA transmission per 1000-colonization-days among the residents during their stay in LTCFs and hospitals were 309 and 113 respectively, while the colonization pressure in LTCFs and hospitals were 210 and 185 per 1000-patient-days respectively. MRSA *spa* type t1081 was the most commonly isolated linage in both LTCF residents (76/121, 62.8%) and hospitalized patients (51/87, 58.6%), while type t4677 was significantly associated with LTCF residents (24/121, 19.8%) compared with hospitalized patients (3/87, 3.4%) (p < 0.001). This suggested continuous transmission of MRSA t4677 among LTCF residents. Also, an inverse linear relationship between MRSA prevalence in LTCFs and the average living area per LTCF resident was observed (Pearson correlation −0.443, p = 0.004), with the odds of patients acquiring MRSA reduced by a factor of 0.90 for each 10 square feet increase in living area.

**Conclusions:**

Our data suggest that MRSA transmission was more serious in LTCFs than in hospitals. Infection control should be focused on LTCFs in order to reduce the burden of MRSA carriers in healthcare settings.

## Background

Methicillin-resistant Staphylococcus aureus (MRSA) has emerged worldwide as an important nosocomial pathogen since 1980s [1]. The transfer of colonized or infected patients between hospitals, and repeated hospital admissions were identified to be the two major causes of nosocomial MRSA acquisition [2,3]. Other risk factors for hospital-acquired MRSA include antibiotic exposure, length of hospital stay, admission to intensive care unit (ICU), colonization pressure, and underlying co-morbidities. Hence, implementation of antimicrobial stewardship program, hand hygiene campaign, and the use of a bundle approach in the adult ICU were highly recommended for the effective control of nosocomial MRSA transmission [4-7].

In Hong Kong, the increasing number of elderly persons urged the need for long term institutional care and frequent hospitalizations. Long term care facilities (LTCFs) providing skilled nursery services for the elderlies in Hong Kong were found to be a major reservoir for MRSA. The prevalence of MRSA carriers among LTCF residents in Hong Kong was 2.8% to 5.1% in 2005 [8,9]. Our recent study showed that 46% of patients with positive MRSA screening upon hospital admission were LTCF residents [10]. Other studies focusing on the prevalence and risk factors for MRSA colonization have also observed that a recent history of hospitalization is an important determinant for MRSA colonization among the population within LTCFs [8,11-18]. However, the relative contribution of LTCFs and hospitals in the degree of MRSA transmission within the healthcare setting is undetermined.

In this study, we analyzed the acquisition of MRSA within LTCFs and hospitals in our locality. The findings of this study may have significant implication for infection control planning and resource allocation in the LTCFs.

## Methods

### Study design

This study compared (i) the prevalence and risk factors of MRSA colonization between the LTCFs and hospitals, and (ii) the incidence of MRSA transmission per 1000-colonization-days among the resident during their stay in LTCFs and hospitals. Furthermore, the transmission of MRSA was analyzed by Staphylococcus protein A (*spa*) typing, and the relationship between MRSA prevalence and living area per LTCF resident was also evaluated in this study.

### Setting and participants

A prospective study was conducted from 1 July to 31 December 2011 to determine the prevalence and acquisition of MRSA among LTCFs and their network hospitals in the Hong Kong West Cluster, which served a population of 0.53 million. LTCFs is a collective term for all long term nursing facilities that provide daily nursing care for their residents including the use of feeding tubes, urinary catheters and other medical devices. The hospital network in our healthcare region included a tertiary referral university-affiliated acute hospital with 1600 beds, 3 extended-care hospitals with a total of 1600 beds, and 1 pediatric hospital. Patients from LTCFs within our healthcare region are admitted to the acute hospital within the region for management. Once stabilized, patients would either be discharged to their original LTCFs or transferred to one of the 3 extended-care hospitals within the regional hospital network before returning to the LTCFs. Community geriatric assessment team, comprising of geriatricians, nurses and allied health professionals, would provide regular on-site visits to the LTCFs within our healthcare region for comprehensive medical follow-up and provide recommendations on infection control measures. In this study, we recruited all residents who agreed to join this study from the 57 LCTFs under the care of the community geriatric assessment team in our healthcare region. Also, we included nasal MRSA screening results of patients admitted to the acute hospital within the study period between 1 July and 31 December 2011 into the study for analysis.

This study protocol has been approved by the Institutional Review Board of the University of Hong Kong/Hospital Authority Hong Kong West Cluster (IRB reference number: UW 11–235).

### Data collection

The objectives of the study and procedures involved were explained to the community geriatric assessment team and LTCFs representatives in our healthcare region. Informed consent was directly obtained from each of the participating residents, or their relatives if the resident was mentally incapacitated. Student nurses were recruited for specimen collection at the LTCFs and hospital admission wards between 1 July and 31 August 2011. They were trained by infection control nurses on the techniques in taking nasal swabs according to a standard protocol as previously described [19]. Patients’ demographic information, history of hospitalization, underlying conditions, and the presence of indwelling devices, wounds or ulcers, were collected from patients’ charts and hospital computer information system. Antimicrobial treatment history within the preceding three months of MRSA screening was also analyzed.

To obtain the number of MRSA carriers among the LTCF residents being hospitalized, MRSA screening from nasal swabs were taken within 24 hours when the the LTCF residents admitted to our acute hospital. Acquisition of MRSA in LTCFs was defined as a negative MRSA screening at the LTCFs between 1 July and 31 August 2011 followed by a positive result upon hospital admission screening. The time interval between the first negative sample collection at the LTCFs and the positive hospital admission screening was recorded. Similarly, to investigate the nosocomial MRSA acquisition among LTCF residents during their hospital stay, nasal swabs for MRSA screening were repeated at the acute and 3 extended-care hospitals before being discharged to the patients’ respective LTCFs. Nosocomial acquisition of MRSA was defined as the conversion of nasal MRSA carriage status from negative to positive during hospitalization. The time interval between hospital admission and discharge was recorded.

The MRSA colonization pressure in different patient groups is estimated using the formula for calculating colonization pressures per 1000-LTCFs resident-days, as described previously [20]. The colonization pressure for LTCF residents was defined as the ratio of MRSA-carrying LTCF resident-days over the total number of LTCF resident-days, while the colonization pressure for hospitalized LTCF residents was defined as the ratio of imported-MRSA hospitalized-days over the total number of hospitalized days during the study. The incidence of MRSA transmission during their stay in LTCFs and in hospitals were measured in terms of MRSA transmission per 1000-colonization-days. The data on the total number of LTCFs resident-days and hospitalized days were collected from the community geriatric assessment team and the hospital record office respectively.

### Data analysis

To determine the differences between patients with probable LTCF-acquired MRSA and probable hospital-acquired MRSA, patients were classified into “LTCFs subgroup” and “hospital subgroup” for further analysis. LTCF residents who had no history of hospitalization in the past 12 months are classified as ‘LTCFs subgroup’, while “hospital subgroup” consisted of non-LTCF patients who were admitted to the acute hospital within the study period. An exposure window of 12 months was selected as the length of monitoring period, since the median carriage of MRSA was found to be 8.5 months after hospital discharge [21], and the 12-months period has also been adopted in other MRSA transmission epidemiology studies [22,23]. The risk factors for MRSA acquisition in the LTCFs subgroup were analyzed, and the MRSA *spa* type distribution between LTCFs and hospital subgroups were compared.

As the general demographic factors showed no significant difference on MRSA acquisition between the two subgroups, we sought for other potential LTCFs specific contributing factor. Hong Kong is a highly populated city with limited land resource and LTCFs are of great demand, therefore LTCFs are often crowded. Thus, we postulate that living area may affect the living standard of the elderly and the average living area in LTCFs may correlate with the hygienic standard of the LTCFs in Hong Kong. The overall MRSA prevalence in LTCFs was compared with the average living area (square feet per person) per resident of different LTCFs. The size of each LTCF was estimated from the government registrations and commercial websites for property trading and anonymous on-site assessment was made by two co-authors to validate the information. The official capacity and occupancy of each LTCF was collected from the community geriatric assessment team. The living area per person was defined as the total area of the LTCF divided by the number of residents at the time of study.

### Microbiological analysis

Swab specimens collected from the study subjects were delivered to the laboratory immediately for inoculation on MRSA chromID culture media (bioMérieux), which was incubated aerobically at 35°C for 48 hours. MRSA colonies were confirmed as previously described [19]. DNA was extracted from *S. aureus* colonies using alkaline lysis method and *spa* typing was performed on the first isolate from each person as previously described [9,10,24]. Repeat sequences were analyzed according to the Ribosomal Differentiation of Micro-organisms (RIDOM) database on *Staphylococcus aureus* (http://www.ridom.de/staphtype) for *spa* typing.

### Statistical analysis

For statistical calculation, the Chi-square test, Fisher’s exact test, *t*-test, or the Mann–Whitney *U*-test was used where appropriate. Pearson correlation was calculated to evaluate the potential linear relationship between overall MRSA prevalence in the LTCFs and the average living area (square feet per person) per LTCF resident. All reported p-values were two-sided. A p-value of <0.05 was considered statistically significant. Computation was performed using the Predictive Analytics Soft Ware (PASW) Version 18.0 (formerly SPSS) for Windows and R 2.14.0.

## Results

### Prevalence and risk factors for MRSA colonization in LTCFs and hospitals

**Figure 1:**
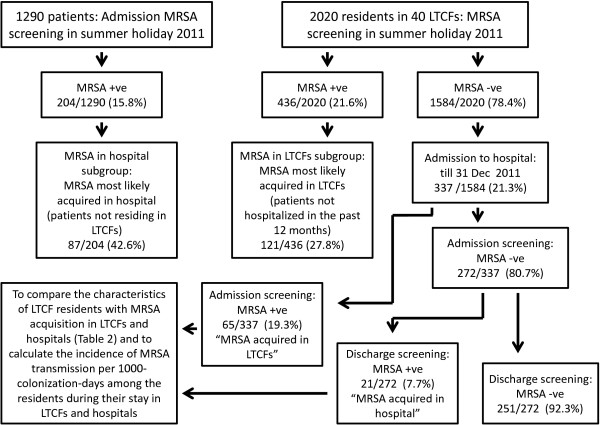
**Overview of the MRSA colonization among LTCF residents and hospitalized LTCF residents in the healthcare region, Hong Kong West.** Note. LTCFs, Long term care facilities.

Of the 57 LTCFs under the coverage of community geriatric assessment service at our healthcare region, 40 (70.2%) LTCFs participated in our study. The LTCF residents had to share toilet facilities. Nursing care was provided by on-site staff but the medical problems were taken care of by the community geriatric assessment team who visits at regular basis. Thirteen percent of residents have in situ feeding tubes, urinary catheters or other medical devices requiring special care. During the study period, 2900 residents lived in these LTCFs, of which 2020 residents (69.7%) consented for the study. Among the 2020 recruited residents, 436 of them (21.6%) were identified to be MRSA positive (Figure 1) through the LTCFs on-site surveillance screening. Compared with the other 1584 recruited residents without MRSA colonization, MRSA carriers had significantly more episodes of hospitalization (72.2% vs 53.7%, p < 0.001) and longer cumulative length of hospital stay in the past 12 months (Additional file 1: Table S1).

During the concurrent period, admission MRSA screening performed for all subjects in the study cohort within 24 hour of admission to the acute hospital. Among the patients from 1290 consecutive hospital admissions, 204 (15.8%) were identified as MRSA-positive (Figure 1). A significantly higher proportion of MRSA-positive patients were admitted directly from the LTCFs (57.4%) comparing to non-LTCF residents (13.6%) [p < 0.001; odd ratio of 8.52 (6.15-11.82)] (Additional file 2: Table S2).

**Figure 2:**
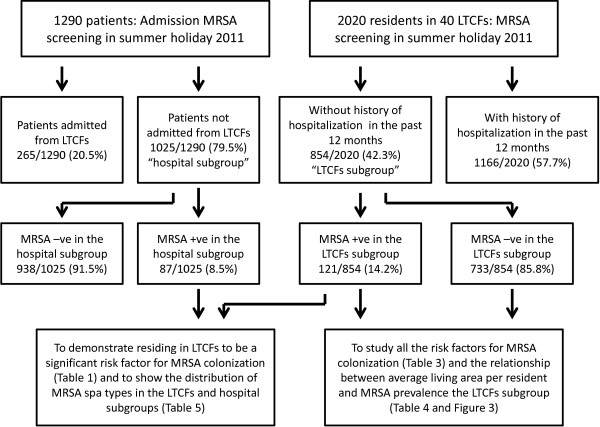
Overview of the logistic of follow up analysis in both LTCF residents and hospitalized patients recruited in our study.

Eight hundred and fifty-four (42.3%) of 2020 LTCF residents with no history of hospitalization in the past 12 months (LTCFs subgroup) and 1025 (79.5%) of 1290 non-LTCF hospitalized patients (hospital subgroup) were selected for further analysis to determine the differences between patients with probable LTCF-acquired MRSA and probable hospital-acquired MRSA. From the LTCFs subgroup, 121 (14.2%) of 854 residents were MRSA carriers, while only 87 (8.5%) of 1025 patients from the hospital subgroup were identified as MRSA carriers (Figure 2). The risk factors for MRSA colonization in the LTCFs and hospital subgroups as determined by logistic regression analysis are shown in Table 1. Residing in LTCFs was shown to be a significant risk factor for MRSA colonization. Moreover, the presence of urinary catheter, chronic cerebral conditions, the use of β-lactam/β-lactamase inhibitors within three months of MRSA screening were also found to be significant risk factors. After adjusting for the confounding factors, the estimated odds for persons having MRSA in LTCFs were 3.4 times higher than those not residing in LTCFs.

**Table 1:** Logistic regression analysis for the estimated probability of detection of MRSA with the following risk factors in the combined LTCFs subgroup and hospital subgroup

	**Estimate**	**Standard error**	**z value**	**p value**
(Intercept)	−3.257	0.189	−17.216	<0.001
Presence of nasogastric tube feeding	0.643	0.286	2.251	0.024
Presence of urinary catheter	1.318	0.259	5.094	<0.001
Chronic cerebral conditions	0.794	0.187	4.247	<0.001
Use of β-lactam/β-lactamase inhibitors within 3 months of MRSA screening	0.860	0.224	3.843	<0.001
Residence in LTCFs	1.218	0.205	5.945	<0.001

LTCFs, long term care facilities.

Hospital subgroup, defined as patients who were not referred from LTCFs; LTCFs subgroup, defined as LTCF residents who had not been hospitalized in the past 12 months.

Note. After adjusting the other confounding factors, the estimated odds for persons having MRSA in LTCFs is exp (1.218) ≈ 3.4 times than those not residing in LTCFs.

### Acquisition of MRSA among residents during their stay in LTCFs and in hospitals

Among the 1584 LTCF residents who were found to be non-MRSA carriers during the on-site surveillance period between 1 July and 31 August 2011, 337 of them (21.3%) were subsequently admitted to the acute hospitals, and were subjected to MRSA admission and discharge screening (Figure 1). Admission screening had identified 65/337 (19.3%) residents to have become MRSA-positive suggesting that they had acquired MRSA in the LTCFs since the time of on-site surveillance. The median time of MRSA detection from surveillance to admission was 77 days (range, 9–181 days). Given that the 436 MRSA-positive residents identified during the on-site surveillance had stayed in the 40 LTCFs for 66,802 days, and the overall 2020 residents had stayed in the LTCFs for 317,752 days during our study period, the colonization pressure of MRSA in LTCFs would be 210 per 1000-resident-days [(MRSA resident-days of 436 MRSA-positive residents was 66,802 days)/(total resident-days of 2020 residents was 317,752 days) × 1000 days]. With the use of these information, the rate of MRSA transmission of the 65 defined LTCFs acquired MRSA was estimated to be 309 MRSA transmissions per 1000-colonization-days among LTCF residents [(65 residents acquired MRSA in LTCFs)/(colonization pressure of 210 per 1000-resident-days) × 1000 days].

During hospitalization, 21 (7.7%) out of 272 the MRSA-negative LTCF residents acquired MRSA. The median time of MRSA detection was 7 days (ranged 1–31 days). Given that the 65 LTCF-acquired MRSA residents stayed in hospital for a total of 396 days, and the 337 non-MRSA carrying LTCF residents, during the on-site surveillance, stayed for 2137 days during our study period, the MRSA colonization pressure for hospitalized LTCF residents was 185 per 1000-patient-days [(imported-MRSA patient-days of 396 days)/(total patient-days of 337 residents of 2137 days) × 1000 days]. Based on this data, we further estimated the rate of MRSA transmission for hospitalized LTCF residents to be 113 MRSA transmissions per 1000-colonization-days [(21 residents acquired MRSA in hospital)/(colonization pressure of 185 per 1000-patient-days) × 1000 days]. The demographic characteristics of persons with MRSA acquisition in LTCFs and hospitals were not significantly difference (Table 2).

**Table 2:** Demographic characteristics of LTCF residents with MRSA acquisition in LTCFs and hospitals

	**MRSA acquisition in LTCFs (n = 65)**	**MRSA acquisition in hospitals (n = 21)**	**p value**
Age (mean ± SD)	85.1 ± 10.2	84.2 ± 10.8	0.744
Sex (male)	24 (36.9%)	8 (38.1%)	0.923
Underlying diseases			
Chronic cerebral conditions ^a^	29 (44.6%)	7 (33.3%)	0.362
Chronic cardiac conditions ^b^	8 (12.3%)	6 (28.6%)	0.079
Chronic pulmonary conditions ^c^	7 (10.8%)	3 (14.3%)	0.662
Chronic renal failure	2 (3.1%)	2 (9.5%)	0.223
Liver cirrhosis	0	0	NA
Diabetes mellitus	11 (16.9%)	5 (23.8%)	0.481
Malignancy	4 (6.2%)	1 (4.8%)	0.813
Presence of			
Nasogastric tube	23 (35.4%)	7 (33.3%)	0.864
Urinary catheter	15 (23.1%)	3 (14.3%)	0.389
Tenckhoff catheter	0	0	NA
Wound or ulcer	1 (1.5%)	2 (9.5%)	0.083
Antibiotics therapy within 3 months of MRSA screening			
Penicillin group	11 (16.9%)	6 (28.6%)	0.244
β-lactam/β-lactamase inhibitors	23 (35.4%)	8 (38.1%)	0.822
Cephalosporin group	7 (10.8%)	3 (14.3%)	0.662
Carbapenem group	0	1 (4.8%)	0.077
Fluoroquinolones	2 (3.1%)	2 (9.5%)	0.223

LTCFs, residential care homes for elderly; MRSA, methicillin-resistant *Staphylococcus aureus*; NA, not applicable; ^a^ chronic cerebral conditions included cerebrovascular accident, dementia, and Parkinson disease; ^b^ chronic cardiac conditions included ischemic heart disease and congestive heart failure; ^c^ chronic pulmonary conditions included chronic bronchitis, chronic obstructive pulmonary disease, and asthma.

### Relationship between MRSA prevalence and living area per LTCFs resident

**Figure 3:**
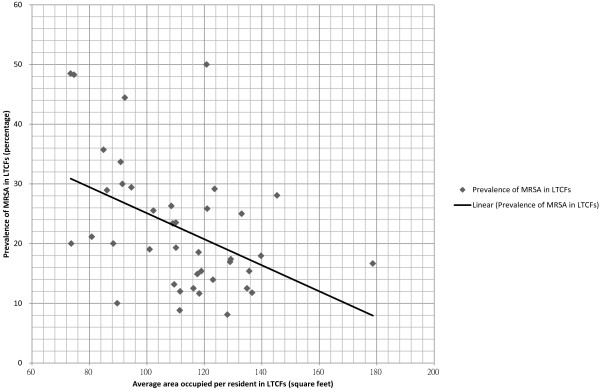
**The relationship between the MRSA prevalence per LTCF and the average living area (square feet per person) per LTCF resident.** Note. Pearson correlation of MRSA prevalence per LTCFs and area (square feet) per person = −0.443 (p = 0.004).

An inverse linear relationship between MRSA prevalence in the LTCFs and average living area (square feet per person) per LTCF resident was found (Figure 3). Pearson correlation of MRSA prevalence per LTCF and living area per resident was −0.443 (p = 0.004). Risk factors for MRSA colonization in the LTCFs subgroup was shown in Table 3. The odds of patients having MRSA reduced by a factor of 0.90 for each 10 square feet increase in area per person when the other risk factors were held constant (Table 4).

**Table 3:** Risk factors for MRSA colonization in the LTCFs subgroup

	**MRSA carrier (n = 121)**	**Non-MRSA carrier (n = 733)**	**p value**
Age (mean ± SD)	84.3 ± 8.9	83.0 ± 10.1	0.144
Sex (male)	35 (28.9%)	246 (33.6%)	0.368
Underlying diseases			
Chronic cerebral conditions ^a^	25 (20.7%)	84 (11.5%)	0.008
Presence of			
Nasogastric tube	13 (10.7%)	39 (5.3%)	0.035
Urinary catheter	5 (4.1%)	5 (0.7%)	0.005
Antibiotics therapy within 3 months of MRSA screening			
β -lactam/β-lactamase inhibitors	6 (5.0%)	15 (2.0%)	0.110
Area (square feet) per person ^b^	108.8 ± 20.1	113.0 ± 19.0	0.031

LTCFs, long term care facilities; LTCFs subgroup, defined as LTCF residents who had not been hospitalized in the past 12 months; SD, standard deviation; ^a^ chronic cerebral conditions included cerebrovascular accident, dementia, and Parkinson disease; ^b^ area per person is defined as total area of the LTCFs over the number of resident occupying at the time of study.

**Table 4:** Logistic regression analysis for the estimated probability of detection of MRSA with the following risk factors in the LTCFs subgroup

	**Estimate**	**Standard error**	**z value**	**p value**
(Intercept)	−0.422	0.578	−0.729	0.46588
Presence of urinary catheter	2.125	0.660	3.219	0.00129
Chronic cerebral conditions	0.742	0.256	2.899	0.00374
Area (square feet) per person ^a^	−0.014	0.006	−2.648	0.0081

LTCFs, long term care facilities; LTCFs subgroup, defined as LTCF residents who had not been hospitalized in the past 12 months; ^a^ area per person is defined as total area of the LTCFs over the number of resident occupying at the time of study.

Note. The odds of a patient having MRSA decreased by exp (−0.14) ≈ 0.90 times with each 10 square feet increase in area (square feet) per person when the other risk factors of presence of urinary catheter and chronic cerebral conditions were held constant.

### Spa type diversity in LTCFs subgroup and hospital subgroup

*Spa* typing was performed for 121 MRSA strains from the LTCFs subgroup and 87 MRSA strains from the hospital subgroup. The *spa* type diversities were significantly different between the two subgroups (Fisher’s exact test, p <0.01) (Table 5). The most common *spa* type was t1081, which constituted 76 (62.8%) of 121 and 51 (58.6%) of 87 MRSA strains from the LTCFs and hospital subgroups respectively. Another *spa* type t4677 was significantly associated with the LTCFs subgroup while t002 was significantly associated with the hospital subgroup. This diversity in *spa* type was also observed among the 337 patients who were MRSA-negative during LTCFs on-site surveillance who were subsequently hospitalized within the study period. The 65 patients (65/337, 19.3%) who were identified to be positive with MRSA at the admission screening had *spa* types which belonged to the LTCFs subgroup, while the 272 (272/337, 80.7%) who were MRSA-negative at admission screening but later became MRSA-positive at discharge screening had *spa* types which belonged to the hospital subgroup.

**Table 5:** **Comparison of MRSA *spa *type in the LTCFs subgroup and hospital subgroup**

**Spa type**	**MRSA in LTCFs subgroup**	**MRSA in hospital subgroup**	**p value**	**Odd ratio**
t002	2 (1.7%)	14 (16.1%)	<0.001	0.09
t032	2 (1.7%)	5 (5.7%)	0.132	0.28
t037	2 (1.7%)	6 (6.9%)	0.070	0.23
t701	8 (6.6%)	1 (1.1%)	0.083	6.05
t1081	76 (62.8%)	51 (58.6%)	0.567	1.19
t4677	24 (19.8%)	3 (3.4%)	<0.001	6.88
Other	7 (5.8%) ^a^	7 (8.0%) ^b^	NA	NA
Total	121 (100%)	87 (100%)	NA	NA

Hospital subgroup, defined as hospitalized patients who were not referred from LTCFs; NA, not applicable; LTCFs, long term care facilities; LTCFs subgroup, defined as LTCF residents who had not been hospitalized in the past 12 months; ^a^ including *spa* types of t121 (2), t012 (1), t1026 (1), t1765 (1), t2536 (1), and t588 (1); ^b^ including spa types of t437 (2), t1250 (1), t1751 (1), t441 (1), t5413 (1), and t9377 (1).

## Discussion

Our study showed that the prevalence of MRSA among LTCFs in Hong Kong had increased substantially from 3-5% to over 20% (436 MRSA positive/2020 residents in 40 LTCFs) (Figure 1) in the recent six years [8,9]. This finding is comparable to those in the United States [25] study but higher than other studies conducted in Germany [11,18], Belgium [12,16], and Spain [15]. Similar to the previous studies, history of hospitalization, chronic comorbidity, indwelling devices, wound or ulcer, and antimicrobial therapy were found to be risk factors for MRSA colonization in our study [11,12,15,16,18]. Moreover, residence in LTCFs and a long cumulative length of hospital stay in the past 12 months were again found to be significant risk factors for MRSA colonization by univariate analysis in our concurrent admission screening [26-30]. Transfer of patients between LTCFs and hospitals creates a vicious cycle which perpetuates MRSA transmission. Hence, it is of great importance to investigate the relative contribution of LTCFs and hospitals in the transmission dynamics of MRSA in the healthcare setting.

Through this study, we identified that acquisition of MRSA among LTCF residents was 3.4 times higher than those patients who did not reside in LTCFs by multivariate analysis. The incidence of MRSA transmission per 1000-colonization-days among LTCF residents was also three times higher than that of the hospitalized LTCF residents, given that the colonization pressure in both LTCFs and hospital were similar. Our findings suggested that MRSA transmission in LTCFs was more severe than that in the hospitals.

While there was no difference in risk factors between patients with LTCFs-acquired and hospital-acquired MRSA, it was noted that the average living area per resident in different LTCFs was an important surrogate marker reflecting the hygienic standard of LTCFs. An inverse linear relationship between MRSA prevalence in the LTCFs and the average living area per resident was found. Provided that the other risk factors were held constant, the odds of patients acquiring MRSA is reduced by a factor of 0.9 for each increment of 10 square feet in living area. To our best knowledge, our study is the first quantitative analysis to demonstrate that living area per person could be a determinant of MRSA prevalence in LTCFs. This finding is particularly relevant for urban cities with a high population density like Hong Kong with an average land price of USD 1000 per square feet. The supply of residential land is limited and living environments are characterized by extremely compact multi-storey apartments [31]. The average living area is an important indicator of the degree of spatial separation, making it a suitable surrogate marker of LTCFs in terms of the overall standard of care, hygiene and infection control. Indeed, overcrowded environment in the correctional facility had been implicated as a contributing factor for MRSA transmission in the United States [32,33].

The *spa* typing results of MRSA strains collected from the LTCFs and hospital subgroups showed that two distinct *spa* type linages, t4677 and t002, were significantly associated with the LTCFs subgroup and the hospital subgroup respectively. Type t002 was commonly found in hospitalized patients in our region and the United States [10,34,35] whereas type t4677 had not been reported in the community or hospital setting previously. This might suggest that t4677 isolates circulated exclusively among LTCF residents in our locality. On the other hand, the high predominance of type t1081 (about 60% of isolates) in both the LTCFs and hospital subgroups could be explained by the intrinsically high transmissibility of t1081 as shown in our previous study [10]. Alternatively, t1081 might have been introduced into our healthcare system in the early years allowing cross-transmission among LTCF residents and hospitalized patients. In fact, t1081 had been predominantly found in our LTCFs in 2005 [9], and t1081, as a member of ST45/Staphylococcus cassette chromosome mec (*SCC*mec) IV or V, was increasingly reported in our hospital isolates from 1995 to 2005 [36].

There are several limitations in our study. Firstly, only 70% of the LTCF residents consented for this study introducing potential bias in subject selection. In addition, we only collected nasal specimens for MRSA screening due to resource limitation. Furthermore, the sensitivity of detection [37] may be compromised in subjects with low microbial load while not on antibiotic therapy [19,38]. Chromogenic agar, however, was used to improve sensitivity and cost-effectiveness [39]. We did not analyze the staffing ratio in LTCFs, which might affect the prevalence of MRSA [40]. As the LTCFs have to satisfy the basic infection control measures required by the government, living area per resident was chosen as an important surrogate marker reflecting the hygienic standard of LTCFs. We did not screen for MRSA carriage among healthcare workers in the LTCFs and hospitals since the benefit of carriage eradication is not established in non-outbreak setting [41], despite a recent study suggesting that both residents and staff were involved in MRSA transmissions [42]. In addition, this is a single season study and may not be applicable to other seasons.

## Conclusion

In summary, we had established the relative importance of LTCFs in the transmission dynamics of MRSA between LTCFs and hospitals in the healthcare setting. More resources should be allocated to improve the infection control measures of LTCFs and further studies are necessary to understand key factors, such as space availability, that lead to high level of MRSA transmission within LTCFs.

## Competing interests

The authors declare that they have no competing interests.

## Authors’ contributions

VCCC and KYY designed, executed and supervised the study. VCCC, JWMT, WCN, DMKC, SSCC, and FHWC coordinated collection of clinical specimens in the long term care facilities. VCCC, JWMT, and JFWC coordinated collection of clinical specimens upon hospital admission. JHKC and MCYY conducted molecular characterization of MRSA strains and validated the area of long term care facilities. ZSYW, KBQP, and HT performed the statistical analysis. VCCC drafted the manuscript. JFWC, SCYW, HT, KLT, PLH and KYY critically reviewed the manuscript. All authors have read and approved the final manuscript.

## Pre-publication history

The pre-publication history for this paper can be accessed here:

http://www.biomedcentral.com/1471-2334/13/205/prepub

## Supplementary Material

Additional file 1: Table S1 Demographic characteristic of 2020 residents from the 40 LTCFs in the Hong Kong West region. Note. LTCFs, long term care facilities; SD, standard deviation. Click here for file

Additional file 2: Table S2 Demographic characteristic of 1290 consecutive patients (from both LTCFs and non-LTCFs) with or without MRSA colonization upon admission. Note. LTCFs, long term care facilities; SD, standard deviation. Click here for file

## References

[B1] Ayliffe GA The progressive intercontinental spread of methicillin-resistant Staphylococcus aureus Clin Infect Dis 1997 24 Suppl 1 S74 79 8994782 10.1093/clinids/24.supplement_1.s74

[B2] Thompson RL Cabezudo I Wenzel RP Epidemiology of nosocomial infections caused by methicillin-resistant Staphylococcus aureus Ann Intern Med 1982 97 3 309 317 10.7326/0003-4819-97-3-309 7114627

[B3] Bradley SF Terpenning MS Ramsey MA Zarins LT Jorgensen KA Sottile WS Schaberg DR Kauffman CA Methicillin-resistant Staphylococcus aureus: colonization and infection in a long-term care facility Ann Intern Med 1991 115 6 417 422 10.7326/0003-4819-115-6-417 1908198

[B4] Cheng VC To KK Li IW Tang BS Chan JF Kwan S Mak R Tai J Ching P Ho PL Antimicrobial stewardship program directed at broad-spectrum intravenous antibiotics prescription in a tertiary hospital Eur J Clin Microbiol Infect Dis 2009 28 12 1447 1456 10.1007/s10096-009-0803-8 19727869

[B5] Cheng VC Tai JW Ho SK Chan JF Hung KN Ho PL Yuen KY Introduction of an electronic monitoring system for monitoring compliance with Moments 1 and 4 of the WHO “My 5 Moments for Hand Hygiene” methodology BMC Infect Dis 2011 11 151 10.1186/1471-2334-11-151 21612666 PMC3129590

[B6] Tai JW Mok ES Ching PT Seto WH Pittet D Nurses and physicians’ perceptions of the importance and impact of healthcare-associated infections and hand hygiene: a multi-center exploratory study in Hong Kong Infection 2009 37 4 320 333 10.1007/s15010-009-8245-x 19636497

[B7] Cheng VC Tai JW Chan WM Lau EH Chan JF To KK Li IW Ho PL Yuen KY Sequential introduction of single room isolation and hand hygiene campaign in the control of methicillin-resistant Staphylococcus aureus in intensive care unit BMC Infect Dis 2010 10 263 10.1186/1471-2334-10-263 20822509 PMC2944349

[B8] Ho PL Wang TK Ching P Mak GC Lai E Yam WC Seto WH Epidemiology and genetic diversity of methicillin-resistant Staphylococcus aureus strains in residential care homes for elderly persons in Hong Kong Infect Control Hosp Epidemiol 2007 28 6 671 678 10.1086/517951 17520539

[B9] Ho PL Lai EL Chow KH Chow LS Yuen KY Yung RW Molecular epidemiology of methicillin-resistant Staphylococcus aureus in residential care homes for the elderly in Hong Kong Diagn Microbiol Infect Dis 2008 61 2 135 142 10.1016/j.diagmicrobio.2007.12.017 18272314

[B10] Cheng VC Chan JF Lau EH Yam WC Ho SK Yau MC Tse EY Wong AC Tai JW Fan ST Studying the transmission dynamics of meticillin-resistant Staphylococcus aureus in Hong Kong using spa typing J Hosp Infect 2011 79 3 206 210 10.1016/j.jhin.2011.03.024 21641082

[B11] von Baum H Schmidt C Svoboda D Bock-Hensley O Wendt C Risk factors for methicillin-resistant Staphylococcus aureus carriage in residents of German nursing homes Infect Control Hosp Epidemiol 2002 23 9 511 515 10.1086/502098 12269448

[B12] Suetens C Niclaes L Jans B Verhaegen J Schuermans A Van Eldere J Vandenbroucke JP Buntinx F Determinants of methicillin-resistant Staphylococcus aureus carriage in nursing homes Age Ageing 2007 36 3 327 330 10.1093/ageing/afm013 17395619

[B13] Barr B Wilcox MH Brady A Parnell P Darby B Tompkins D Prevalence of methicillin-resistant Staphylococcus aureus colonization among older residents of care homes in the United Kingdom Infect Control Hosp Epidemiol 2007 28 7 853 859 10.1086/516795 17564989

[B14] Brugnaro P Fedeli U Pellizzer G Buonfrate D Rassu M Boldrin C Parisi SG Grossato A Palu G Spolaore P Clustering and risk factors of methicillin-resistant Staphylococcus aureus carriage in two Italian long-term care facilities Infection 2009 37 3 216 221 10.1007/s15010-008-8165-1 19148574

[B15] Mariscal D Dominguez MA Perez JL Segura F Pujol M Ruiz De Gopegui E Manzur A Gavalda L Prevalence of methicillin-resistant Staphylococcus aureus and factors associated with colonization among residents in community long-term-care facilities in Spain Clin Microbiol Infect 2008 14 9 867 872 10.1111/j.1469-0691.2008.02060.x 18844688

[B16] Denis O Jans B Deplano A Nonhoff C De Ryck R Suetens C Struelens MJ Epidemiology of methicillin-resistant Staphylococcus aureus (MRSA) among residents of nursing homes in Belgium J Antimicrob Chemother 2009 64 6 1299 1306 10.1093/jac/dkp345 19808236

[B17] Lasseter G Charlett A Lewis D Donald I Howell-Jones R McNulty CA Staphylococcus aureus carriage in care homes: identification of risk factors, including the role of dementia Epidemiol Infect 2010 138 5 686 696 10.1017/S0950268810000233 20149266

[B18] Pfingsten-Wurzburg S Pieper DH Bautsch W Probst-Kepper M Prevalence and molecular epidemiology of meticillin-resistant Staphylococcus aureus in nursing home residents in northern Germany J Hosp Infect 2011 78 2 108 112 10.1016/j.jhin.2011.02.011 21481969

[B19] Cheng VC Li IW Wu AK Tang BS Ng KH To KK Tse H Que TL Ho PL Yuen KY Effect of antibiotics on the bacterial load of meticillin-resistant Staphylococcus aureus colonisation in anterior nares J Hosp Infect 2008 70 1 27 34 10.1016/j.jhin.2008.05.019 18632184

[B20] Eveillard M Lancien E Hidri N Barnaud G Gaba S Benlolo JA Joly-Guillou ML Estimation of methicillin-resistant Staphylococcus aureus transmission by considering colonization pressure at the time of hospital admission J Hosp Infect 2005 60 1 27 31 10.1016/j.jhin.2004.10.008 15823653

[B21] Scanvic A Denic L Gaillon S Giry P Andremont A Lucet JC Duration of colonization by methicillin-resistant Staphylococcus aureus after hospital discharge and risk factors for prolonged carriage Clin Infect Dis 2001 32 10 1393 1398 10.1086/320151 11317238

[B22] Harbarth S Sax H Fankhauser-Rodriguez C Schrenzel J Agostinho A Pittet D Evaluating the probability of previously unknown carriage of MRSA at hospital admission Am J Med 2006 275 3 e215 223 119 10.1016/j.amjmed.2005.04.042 16490475

[B23] Furuno JP McGregor JC Harris AD Johnson JA Johnson JK Langenberg P Venezia RA Finkelstein J Smith DL Strauss SM Identifying groups at high risk for carriage of antibiotic-resistant bacteria Arch Intern Med 2006 166 5 580 585 10.1001/archinte.166.5.580 16534047

[B24] Ho PL Chuang SK Choi YF Lee RA Lit AC Ng TK Que TL Shek KC Tong HK Tse CW Community-associated methicillin-resistant and methicillin-sensitive Staphylococcus aureus: skin and soft tissue infections in Hong Kong Diagn Microbiol Infect Dis 2008 61 3 245 250 10.1016/j.diagmicrobio.2007.12.015 18272316

[B25] Reynolds C Quan V Kim D Peterson E Dunn J Whealon M Terpstra L Meyers H Cheung M Lee B Methicillin-resistant Staphylococcus aureus (MRSA) carriage in 10 nursing homes in Orange County California Infect Control Hosp Epidemiol 2011 32 1 91 93 10.1086/657637 21087124

[B26] Samad A Banerjee D Carbarns N Ghosh S Prevalence of methicillin-resistant Staphylococcus aureus colonization in surgical patients, on admission to a Welsh hospital J Hosp Infect 2002 51 1 43 46 10.1053/jhin.2002.1182 12009819

[B27] Gopal Rao G Michalczyk P Nayeem N Walker G Wigmore L Prevalence and risk factors for meticillin-resistant Staphylococcus aureus in adult emergency admissions–a case for screening all patients? J Hosp Infect 2007 66 1 15 21 10.1016/j.jhin.2007.01.013 17376560

[B28] Hidron AI Kourbatova EV Halvosa JS Terrell BJ McDougal LK Tenover FC Blumberg HM King MD Risk factors for colonization with methicillin-resistant Staphylococcus aureus (MRSA) in patients admitted to an urban hospital: emergence of community-associated MRSA nasal carriage Clin Infect Dis 2005 41 2 159 166 10.1086/430910 15983910

[B29] Haley CC Mittal D Laviolette A Jannapureddy S Parvez N Haley RW Methicillin-resistant Staphylococcus aureus infection or colonization present at hospital admission: multivariable risk factor screening to increase efficiency of surveillance culturing J Clin Microbiol 2007 45 9 3031 3038 10.1128/JCM.00315-07 17626171 PMC2045295

[B30] Eveillard M Ernst C Cuviller S Lescure FX Malpaux M Defouilloy I Gresanleux M Duboisset M Lienard J Eb F Prevalence of methicillin-resistant Staphylococcus aureus carriage at the time of admission in two acute geriatric wards J Hosp Infect 2002 50 2 122 126 10.1053/jhin.2001.1152 11846539

[B31] Chan EY Kim JH Griffiths SM Lau JT Yu I Does living density matter for nonfatal unintentional home injury in Asian urban settings? Evidence from Hong Kong J Urban Health 2009 86 6 872 886 10.1007/s11524-009-9389-9 19636708 PMC2791815

[B32] Baillargeon J Kelley MF Leach CT Baillargeon G Pollock BH Methicillin-resistant Staphylococcus aureus infection in the Texas prison system Clin Infect Dis 2004 38 9 e92 95 10.1086/383146 15127360

[B33] Pan ES Diep BA Carleton HA Charlebois ED Sensabaugh GF Haller BL Perdreau-Remington F Increasing prevalence of methicillin-resistant Staphylococcus aureus infection in California jails Clin Infect Dis 2003 37 10 1384 1388 10.1086/379019 14583874

[B34] Tenover FC Tickler IA Goering RV Kreiswirth BN Mediavilla JR Persing DH Characterization of nares and blood culture isolates of methicillin-resistant staphylococcus aureus from patients in United States hospitals Antimicrob Agents Chemother 2011 56 3 1324 1330 22155818 10.1128/AAC.05804-11 PMC3294931

[B35] Ho CM Ho MW Lee CY Tien N Lu JJ Clonal spreading of methicillin-resistant SCCmec Staphylococcus aureus with specific spa and dru types in central Taiwan Eur J Clin Microbiol Infect Dis 2012 31 4 499 504 10.1007/s10096-011-1338-3 21789606

[B36] Ho PL Chow KH Lo PY Lee KF Lai EL Changes in the epidemiology of methicillin-resistant Staphylococcus aureus associated with spread of the ST45 lineage in Hong Kong Diagn Microbiol Infect Dis 2009 64 2 131 137 10.1016/j.diagmicrobio.2009.01.030 19345036

[B37] Sewell DL Potter SA Jacobson CM Strausbaugh LJ Ward TT Sensitivity of surveillance cultures for the detection of methicillin-resistant Staphylococcus aureus in a nursing-home-care unit Diagn Microbiol Infect Dis 1993 17 1 53 56 10.1016/0732-8893(93)90070-N 8359006

[B38] Cheng VC Chan JF To KK Tai JW Ho PL Detection of community-associated MRSA as a result of the unmasking effect of antibiotic treatment J Hosp Infect 2009 72 3 273 274 10.1016/j.jhin.2009.03.011 19446364

[B39] Wassenberg MW Kluytmans JA Box AT Bosboom RW Buiting AG van Elzakker EP Melchers WJ van Rijen MM Thijsen SF Troelstra A Rapid screening of methicillin-resistant Staphylococcus aureus using PCR and chromogenic agar: a prospective study to evaluate costs and effects Clin Microbiol Infect 2010 16 12 1754 1761 10.1111/j.1469-0691.2010.03210.x 20219077

[B40] Loeb MB Craven S McGeer AJ Simor AE Bradley SF Low DE Armstrong-Evans M Moss LA Walter SD Risk factors for resistance to antimicrobial agents among nursing home residents Am J Epidemiol 2003 157 1 40 47 10.1093/aje/kwf173 12505889

[B41] Hawkins G Stewart S Blatchford O Reilly J Should healthcare workers be screened routinely for meticillin-resistant Staphylococcus aureus? A review of the evidence J Hosp Infect 2011 77 4 285 289 10.1016/j.jhin.2010.09.038 21292349

[B42] Schwaber MJ Masarwa S Navon-Venezia S Kandlik Y Chmelnitsky I Smollan G Glick R Neria G Carmeli Y High prevalence of methicillin-resistant Staphylococcus aureus among residents and staff of long-term care facilities, involving joint and parallel evolution Clin Infect Dis 2011 53 9 910 913 10.1093/cid/cir607 21984272

