# Supplementary material for: Transmission of methicillin-resistant staphylococcus aureus in the long term care facilities in Hong Kong
Source: BMC Infect Dis. 2013 May 6;13:205. doi: 10.1186/1471-2334-13-205 (PMC3651730; doi:10.1186/1471-2334-13-205)
Supplement: Additional file 1: Table S1 — Demographic characteristic of 2020 residents from the 40 LTCFs in the Hong Kong West region. Note. LTCFs, long term care facilities; SD, standard deviation. [file 1471-2334-13-205-S1.doc]

**Supplementary Table 1.** Demographic characteristic of 2020 residents from the 40 LTCFs in the Hong Kong West region

|  | MRSA carrier (n=436) | Non-MRSA carrier (n=1584) | p value |
| --- | --- | --- | --- |
| Age (mean ± SD) | 84.1 ± 9.2 | 83.5 ± 9.4 | 0.255 |
| Sex (male) | 165 (37.8%) | 563 (35.5%) | 0.376 |
| History of hospital admission in the past 12 months | 315 (72.2%) | 851 (53.7%) | <0.001 |
| Cumulative day of hospitalization in the past 12 months | 18.8 ± 29.5 | 9.8 ± 19.2 | <0.001 |
| Underlying diseases |  |  |  |
| Chronic cerebral conditions | 140 (32.1) | 341 (21.5%) | <0.001 |
| Chronic cardiac conditions | 69 (15.8%) | 172 (10.9%) | 0.005 |
| Chronic pulmonary conditions | 35 (8.0%) | 76 (4.8%) | 0.009 |
| Chronic renal failure | 19 (4.4%) | 35 (2.2%) | 0.014 |
| Liver cirrhosis | 1 (0.2%) | 10 (0.6%) | 0.313 |
| Diabetes mellitus | 80 (18.3%) | 196 (12.4%) | 0.001 |
| Malignancy | 26 (6.0%) | 48 (3.0%) | 0.004 |
| Presence of |  |  |  |
| Nasogastric tube | 83 (19.0%) | 193 (12.2%) | <0.001 |
| Urinary catheter | 80 (18.3%) | 153 (9.7%) | <0.001 |
| Tenckhoff catheter | 0 | 4 (0.3%) | 0.294 |
| Wound or ulcer | 41 (9.4%) | 39 (2.5%) | <0.001 |
| Received antibiotics 3 months before admission (yes / no) | 160 (36.7%) | 307 (19.4%) | <0.001 |
| Penicillin group | 41 (9.4%) | 88 (5.6%) | 0.004 |
| β-lactam / β-lactamase inhibitors | 125 (28.7%) | 209 (13.2%) | <0.001 |
| Cephalosporin group | 33 (7.6%) | 77 (4.9%) | 0.027 |
| Carbapenem groups | 8 (1.8%) | 6 (0.4%) | 0.001 |
| Fluoroquinolones | 25 (5.7%) | 30 (1.9%) | <0.001 |

Note. SD, standard deviation; LTCFs, residential care home for elderly
