# Supplementary material for: Transmission of methicillin-resistant staphylococcus aureus in the long term care facilities in Hong Kong
Source: BMC Infect Dis. 2013 May 6;13:205. doi: 10.1186/1471-2334-13-205 (PMC3651730; doi:10.1186/1471-2334-13-205)
Supplement: Additional file 2: Table S2 — Demographic characteristic of 1290 consecutive patients (from both LTCFs and non-LTCFs) with or without MRSA colonization upon admission. Note. LTCFs, long term care facilities; SD, standard deviation. [file 1471-2334-13-205-S2.doc]

**Supplementary Table 2.** Demographic characteristic of 1290 consecutive patients (from both LTCFs and non-LTCFs) with or without MRSA colonization upon admission

|  | MRSA carrier (n=204) | Non-MRSA carrier (n=1086) | p value |
| --- | --- | --- | --- |
| Age (mean ± SD) | 80.3 ± 13.3 | 70.6 ± 17.2 | <0.001 |
| Sex (male) | 84 (41.2%) | 537 (49.4%) | 0.03 |
| Residing in LTCFs | 117 (57.4%) | 148 (13.6%) | <0.001 |
| History of hospital admission in the past 12 months | 166 (81.4%) | 643 (59.2%) | <0.001 |
| Cumulative day of hospitalization in the past 12 months | 31.0 ± 34.1 | 19.9 ± 31.7 | <0.001 |
| Underlying diseases |  |  |  |
| Chronic cerebral conditions | 93 (45.6%) | 194 (17.9%) | <0.001 |
| Chronic cardiac conditions | 58 (28.4%) | 252 (23.2%) | 0.109 |
| Chronic pulmonary conditions | 37 (18.1%) | 103 (9.5%) | <0.001 |
| Chronic renal failure | 20 (9.8%) | 88 (8.1%) | 0.421 |
| Liver cirrhosis | 1 (0.5%) | 22 (2.0%) | 0.128 |
| Diabetes mellitus | 65 (31.9%) | 254 (23.4%) | 0.010 |
| Malignancy | 28 (13.7%) | 170 (15.7%) | 0.483 |
| Presence of |  |  |  |
| Nasogastric tube | 55 (27.0%) | 32 (2.9%) | <0.001 |
| Urinary catheter | 61 (29.9%) | 103 (9.5%) | <0.001 |
| Tenckhoff catheter | 1 (0.5%) | 13 (1.2%) | 0.371 |
| Wound or ulcer | 28 (13.7%) | 52 (4.8%) | <0.001 |
| Received antibiotics 3 months before admission (yes / no) |  |  |  |
| Penicillin group | 23 (11.3%) | 53 (4.9%) | <0.001 |
| β-lactam / β-lactamase inhibitors | 148 (72.5%) | 458 (42.2%) | <0.001 |
| Cephalosporin group | 43 (21.1%) | 177 (16.3%) | 0.096 |
| Carbapenem groups | 9 (4.4%) | 58 (5.3%) | 0.583 |
| Fluoroquinolones | 22 (10.8%) | 83 (7.6%) | 0.132 |

Note. SD, standard deviation; LTCFs, Long term care facilities
